# Platelet-Rich Plasma (PRP) for Acute Muscle Injury: A Systematic Review

**DOI:** 10.1371/journal.pone.0090538

**Published:** 2014-02-28

**Authors:** Mohamad Shariff A. Hamid, Ashril Yusof, Mohamed Razif Mohamed Ali

**Affiliations:** 1 Unit of Sports Medicine, Faculty of Medicine, University of Malaya, Kuala Lumpur, Malaysia; 2 Sports Centre, University of Malaya, Kuala Lumpur, Malaysia; 3 Department of Orthopaedic Surgery, Faculty of Medicine, University of Malaya, Kuala Lumpur, Malaysia; University of Pittsburgh, United States of America

## Abstract

**Introduction:**

Acute muscle injury is one of the commonest injuries that often result in loss of training and competition time. The best management for muscle injury has not been identified. Sports medicine practitioners used several approaches in attempt to accelerate time to recovery from muscle injury. More recently growing interest focussed on autologous blood product injection.

**Methods:**

A literature search was conducted systematically using OvidMEDLINE, PubMed, EMBASE, SPORTDiscus and CINAHL databases to retrieve articles published until December 2012. Controlled trials and controlled laboratory studies comparing different strategies to promote early recovery of muscle injury were included. The methodological quality of studies was assessed.

**Results:**

There are limited studies on the effects of PRP therapy for muscle injury. Three *in vivo* laboratory studies and one pilot human study were reviewed. The laboratory studies reported histological evidence on significant acceleration of muscle healing in animals treated with autologous conditioned serum (ACS), platelet-rich plasma (PRP) and platelet rich fibrin matrix (PRFM). A pilot human study found athletes treated with repeated ACS injection recovers significantly faster than retrospective controls.

**Conclusion:**

Several *in vivo* laboratory studies suggest beneficial effects of ACS, PRP and PRFM in accelerating muscle recovery. Evidence to suggest similar effects on humans is however limited, as valuable information from robust human controlled trials is still not available at this moment. Hence, more studies of satisfactory methodological quality with platelet-rich plasma interventions on muscle injury are justified.

## Introduction

Acute muscle injury is one of the commonest types of injury seen in athletes [1]–[3]. This injury often results in loss of training and competition time [4]–[9]. Despite of its frequent occurrence, the best treatment for muscle injury is yet to be identified. Current mode of management usually involves rest, ice, compression and elevation especially in the early stage following injury [10]–[14]. Other modalities includes anti-inflammatory medications (pain killers), rehabilitation exercise programs, electrotherapeutic modalities, hyperbaric oxygen therapy, and prolotherapy injections [15]–[18]. However, clinical evidence to support the use of these modalities is limited.

More recently, injection of autologous platelet-rich plasma (PRP) has gained a lot of attention in the treatment of sports injuries including acute muscle injury [19]–[24]. The rationale for the use of PRP is the belief that the additional growth factors released by platelets would augment the natural healing process. Despite its increasing popularity as a treatment for muscle injury, there is a growing debate regarding PRP clinical efficacy [25]–[27]. The objective of this review is to explore the current literature on the effectiveness of PRP treatment for acute muscle injury.

## Methods

### Data Sources

Studies were searched electronically using the following databases: OvidMEDLINE, PubMed, EMBASE, SPORTDiscus and CINAHL. The reference lists of review articles and included studies were hand searched for other potentially eligible studies using the same selection criteria as described. Published systematic reviews on PRP were used as a source of randomized controlled trials. Peer-reviewed published articles until December 2012 were used. In view of limited resources for translation, only articles published in English were considered. No attempts were made to contact authors for additional information, however, cross-referencing on related previously published study is performed to obtain additional information. The search strategy used for OvidMEDLINE is displayed in Table 1. Comparable searches were made for the other databases. In addition, a search through a local library for archived articles from the South East Asian region using similar selection criteria was also conducted.

**Table 1:** Search strategy for OvidMEDLINE.

	Dates: Jan 1946– Dec 2012	Result
1	Exp Platelet-Rich Plasma/	1338
2	platelet rich fibrin matrix.mp	16
3	autologous conditioned serum.mp	22
4	platelet concentrate.mp	602
5	platelet gel.mp	173
6	autologous growth factors.mp	52
7	preparation rich in growth factors.mp	14
8	platelet releasate.mp	101
9	platelet lysate.mp	281
10	leucocyte platelet rich plasma.mp	1
11	platelet leucocyte rich plasma.mp	0
12	muscle injury.mp	1738
13	1 or 2 or 3 or 4 or 5 or 6 or 7 or 8 or 9 or 10 or 11	2417
14	12 and 13	5

### Study Selection

This qualitative systematic review includes the description of the criteria for study selection and the search methods for identification of studies, detailed qualitative synthesis of the selected studies and the discussion of the findings from this review. The search was conducted according to the Preferred Reporting Items for Systematic reviews and Meta-Analyses (PRISMA) guideline [28]. The process of this search method included describing the data sources, search strategy, data extraction and quality assessment. The supporting PRISMA checklist is available as supporting information; See Checklist S1.

All controlled trials and controlled laboratory studies were considered in this review. Studies that were conducted on adults (≥18 years) diagnosed with acute muscle injury and using interventions to promote early recovery were included. The interventions could include one or combination of (1) rehabilitation program and (2) autologous blood products including PRP. No restrictions were defined regarding the type and contents of the control group. The interventions could be compared with no intervention control or minimal intervention control group. The primary outcome measure in the selected studies was the information on the duration to achieve full recovery or duration to return-to-play (DRP).

### Data Extraction

The titles and abstracts of all studies retrieved from the search were reviewed following criteria for study selection to decide if the full-text manuscripts were required for further evaluation. Each full-text manuscript were evaluated systematically according to the study’s, (1) objective/s, (2) characteristics of the study (study design, participants, age and sample size), (3) contents of intervention (intervention strategies, intervention provider, length of intervention and follow-up contacts), (4) targeted outcome/s, and (5) major findings. The outcomes extracted from the selected study were not combined and re-analysed due to the nature of this qualitative systematic review.

Each selected article was further evaluated for the methodological quality. Two investigators independently graded the methodological quality of each eligible article using the Physiotherapy Evidence Database Scale (PEDro) for randomized controlled trials [29]. The PEDro scale is an 11-point list using yes and no responses. The first statement pertains to the external validity of the study and is not included to compute the final score. The total score ranges from 0 to 10 and represents the number of positive answers on questions 2–11. The PEDro items are shown in Table 2. The reliability of PEDro scale is fair to good [30]. A PEDro score of ≥6 was considered to represent a high quality study, whereas a score of ≤5 represented a low quality study [31]. Differences in opinion on any PEDro item score were resolved through discussion until a consensus was reached.

**Table 2:** PEDro scale.

No.	Criteria	No	Yes	Where
1.	Eligibility criteria were specified	□	□	
2.	Subjects were randomly allocated to groups (in a crossover study, subjects were randomly allocated an order inwhich treatments were received)	□	□	
3.	Allocation was concealed	□	□	
4.	The groups were similar at baseline regarding the most important prognostic indicators	□	□	
5.	There was blinding of all subjects	□	□	
6.	There was blinding of all therapist who administered the therapy	□	□	
7.	There was blinding of all assessors who measured at least one key outcome	□	□	
8.	Measure of at least one key outcome were obtained from more than 85% of the subjects initially allocated to groups	□	□	
9.	All subjects for whom outcome measures were available received the treatment or control condition as allocated or,where this was not the case, data for at least one key outcome was analysed by “intention to treat”	□	□	
10.	The results of between-group statistical comparisons were reported for at least one key outcome	□	□	
11.	The study provides both point measures and measures of variability for at least one key outcome	□	□	

## Results

**Figure 1:**
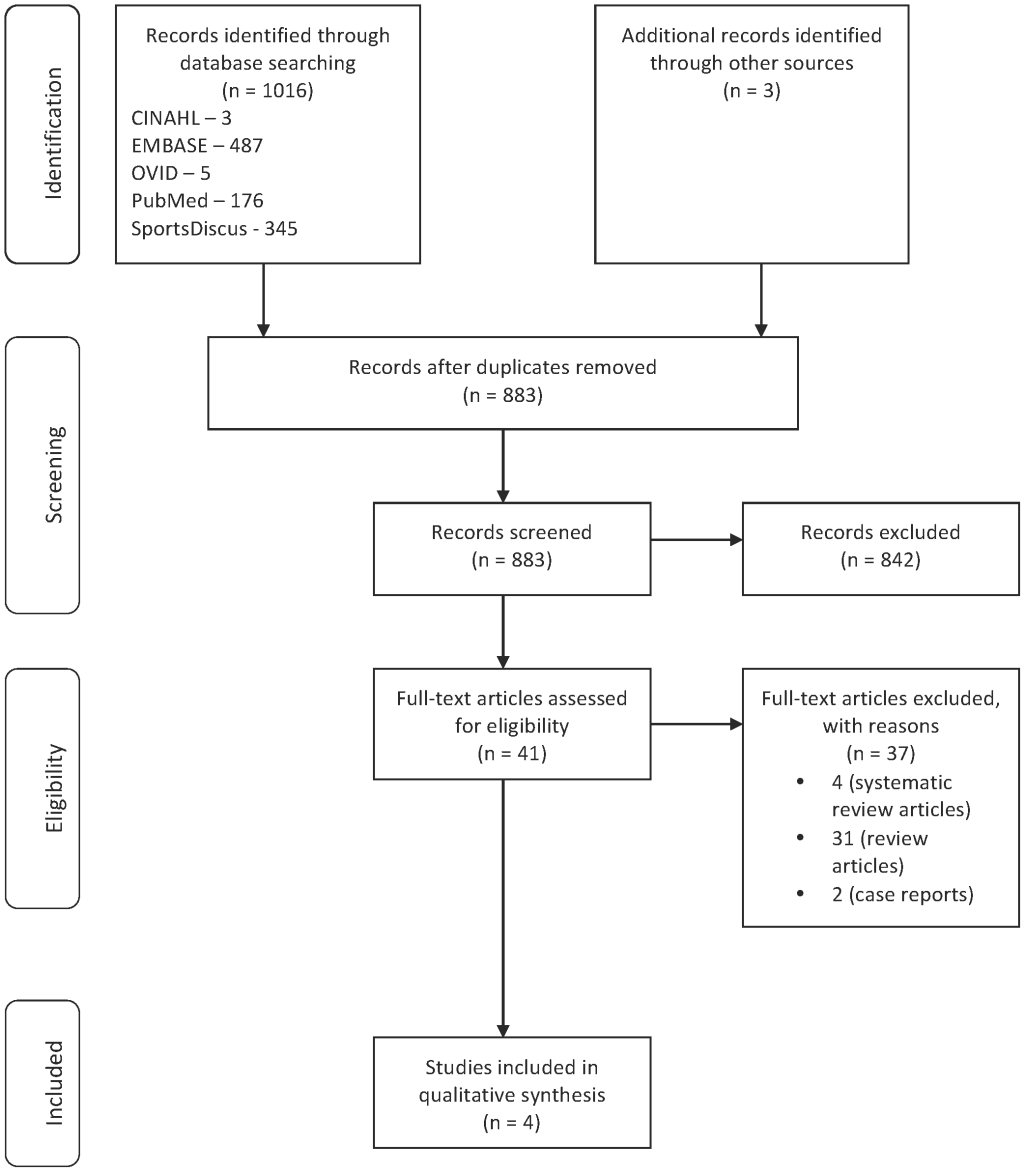
PRISMA flow diagram for study selection.

The initial search identified 1016 potential articles from the databases search and another 3 were found through cross-referencing. After removing duplicates, 883 articles were assessed based on titles and abstracts against the selection criteria. A total of 842 articles were excluded because the studies were not on autologous PRP and muscle injury. Of the 41 full-text articles retrieved for further evaluation, only four articles were included in the final qualitative synthesis. The remaining 37 articles were excluded because 35 of these articles were review articles (including systematic reviews) while the remaining two were case reports. Figure 1 describes the PRISMA flow diagram for the study selection. All articles were published after the year 2004, and in English language. Table 3, describes the characteristics of selected studies. Out of the final four studies selected for the review, there was only one controlled trial (CT) [32], while the remaining three were *in vivo* laboratory studies [32]–[34]. Consequently the discussion on human clinical trial and laboratory studies was conducted separately.

**Table 3:** Characteristics of selected studies.

Studies	Study design/target population	Treatment	Type ofinjury/location	Outcome measures	Results
**Clinical study**					
Wright-Carpenter*et al* [34]	Pilot controlled-clinicaltrial: n = 16(ACS);n = 11(controls)/professional sportsmen	ACS (combined with LA)injections vs. Traumeel/Actovegin (controls)injections bothrepeated every secondday	2^nd^ degree muscle tears(MRI confirmed), mostinjuries to the hamstringand adductors musclegroup	Time to recovery basedon the participant’ssubjective judgementof readiness	Time to recovery was significantly shorter in ACS (16.6 days) than control groups (22.3 days). No side effects of treatment
**Laboratory studies**					
Wright-Carpenter*et al* [30]	Controlled laboratorystudy: n = 39(ACS),n = 39(control)/syngeneic C57B1/6mice	ACS vs saline injectionsat 2, 24 & 48 hrs aftercontusion impact	Iatrogenic contusioninjury of thegastrocnemius muscles	Regenerationquantification: Activatedsatellite cell and sizeof regeneratingmyofibers	Significant increased in satellite cells activation at 30 & 48 hrs after injury. Larger diameter of CN cells in ACS group after 1 week
Hammond *et al* [31]	Controlled laboratorystudy: n = 72 Adultmale Sprague-Dwaleyrats	No treatment vs PRP vsPPP injections at Day 0,3, 5 & 7 after inducedeccentric injury	Iatrogenic eccentricinjury of tibialis anteriormuscle: Single repetition(large strain) andmultiple repetition(small strain)	Functional recovery:Maximal isometriccontraction and Muscleregeneration: MyoD &myogenin markers	Large strain injury: PRP significantly improve contractile function at Day 3. While, Small strain injury: PRP significantly improved contractile function at Day 7 and 14. Full recovery at Day 14. Muscle regeneration MyoD and myogenin significantly increased in PRP treated. Significantly higher number of CN cells in PRP group compared with PPP and no treatment
Gigante *et al* [32]	Controlled lab study:n = 20 male Wistar rats	PRFM vs No treatment(control). Randomallocation.	Iatrogenic tear (pincertechnique) bilaterallongissimus muscle	Blind assessment:Vascularization & muscleregeneration andinflammation & fibrosis	PRFM group: More muscle regeneration at D5 & D10 and more neovascularization at D40 & D60. Less fibrosis at D10. No differences in inflammation

ACS- autologous conditioned serum; LA-local anaesthetic; MRI-magnetic resonance imaging; CN-centronucleated.

### Clinical Study

This pilot CT study was conducted in a clinic setting (Clinic for Sports Medicine & Orthopaedic). The participants in this study were professional sportsmen diagnosed with “moderate strains” (second degree). The diagnosis of injury was based on clinical assessment [35] as well as magnetic resonance imaging (MRI) examinations (detection of bleeding of the involved muscle). The mean age of participants and other demographics in the both groups were not available for comparisons. The intervention used in this study was intra-lesional injection of 2.5 ml autologous conditioned serum (ACS) combined with 2.5 ml of saline. The method of ACS preparation was well described. The intra-lesional injection was guided only through palpation of the affected area. Prior to administration of ACS, 5 ml of local anaesthetic (Meaverin 0.5%) was injected in portion of 1 ml to minimise the tonus of the injured muscle. The ACS injections started two days after diagnosis and were repeated every second day until full recovery. The mean number of ACS injection throughout the study was 5.4 injections per patient. Interestingly the control group in this study was a retrospective analysis of 11 patients who had been treated with local injection of Actovegin/Traumeel (3∶2) combination therapy. Actovegin is a deproteinised dialysate of bovine blood, while Traumeel is a homeopathic formulation containing both botanical and mineral ingredients. It is purported to suppress the release of inflammatory mediators and stimulates the release of anti-inflammatory cytokines. Local injection of Actovegin/Traumeel is considered a standard treatment of muscle strain in this centre [32]. The principles of administration were the same as those in the ACS group. The mean number of treatments with Actovegin/Traumeel per patient was 8.3. Participants in both groups underwent the same rehabilitation program and were given oral antipholgistics. The frequency and dosages of these treatments were not specified. The severity of muscle tears was similar between intervention and control groups. However, the extent (size) of the injured area was not documented. The ACS prepared was analysed to determine the types and quantity of growth factors present with ELISA tests. The ACS contains higher concentration of FGF-2 (750%), IL-1Ra (600%), HGF (35%) and TGF-β (31%) compared to levels in the serum [36].

The main outcome measured was the time required to resume full sporting activities. Return to full sporting activities was based on participant’s subjective impression of readiness to resume activities and physiotherapist’s standard examination, including restoration of muscle strength to at least 90% of that of the unaffected limb. The isokinetic strength test described however was not performed, as researchers were concern with the risk of re-injury during testing. The mean recovery time for participants in the ACS group (16.6 days) was significantly shorter compared to the control group (22.3 days.). In addition, MRI scans taken at 16 days in both groups demonstrated faster regression of the oedema/bleeding in the ACS group. Both treatments were considered safe, as there were no local or systemic side effects reported [36].

### *In vivo* Laboratory Studies

All studies were controlled animal studies conducted on different species of syngeneic rodents [32]–[34]. Studies differ in their methods of inducing muscle injuries. In one study muscle contusion on the animal’s gastrocnemius muscle was induced by dropping a stainless steel ball on the animal’s hind limb from the height of 100 cm [30]. Whereas Hammond *et al.* induced eccentric muscle injury over the tibialis anterior muscle by superimposing a single or multiple eccentric muscle contraction onto a maximally isometric contracted muscle [33]. Gigante *et al.* produced bilateral muscle tears on the longissimus dorsi muscle using a standard pincer technique. As myogenesis relies upon satellite cells activation, proliferation, differentiation, fusion with existing damaged muscle and maturation (increased myofiber diameter) [10]. Accordingly, all studies quantified amount of muscle regeneration via immuno-histochemical staining as one of their outcome measures. Wright-Carpenter *et al*. used Ki-67 labelled antibody as marker of satellite cells proliferation [30]. Whereas Hammond *et al.* and Gigante *et al.* both assayed the level of MyoD and Myogenin as markers of muscle regeneration [33]–[34]. In addition, both Wright-Carpenter *et al.* and Hammond *et al.* also quantified the percentages of centrally nucleated fibres (CNFs) presence in the injured area as an additional measure of myogenesis. Only one study assessed the functional recovery of injured muscle using maximal isometric torque test on the tibialis anterior muscle [33].

### Characteristics of Interventions

The intervention used in each study varies markedly. Using a method originally developed for human blood, Wright-Carpenter *et al.* utilised blood from 20 syngenic mice to produced autologous conditioned serum (ACS) [37]. Animals in the intervention group received 10 µl of ACS at days 0, 3, 5 and 7. While controls received 10 µl of saline injection administered at similar intervals [32]. Enzyme linked immunoassay (ELISA) tests demonstrated higher level of FGF-2 (460%) and TGF-β1 (82%) in the ACS than serum. Hammond *et al.* used 20 ml of blood collected from five adult male Sprague-Dawley rats to produce autologous platelet-rich plasma (PRP) using a commercial kit. The autologous PRP was later conditioned using high-frequency ultrasound to lyse the platelets and release the growth factors thus enriching the PRP prior to injection. The ELISA tests demonstrated significantly higher concentration of PDGF and IGF-1 in PRP compared to platelet-poor plasma (PPP). The level of PDGF and IGF-1 further increased (a 5-fold in PDGF and a 27% in IGF-1) upon conditioning. The intervention group was injected with 100 µl of PRP into the injured tibialis anterior while the controls received platelet-poor plasma or no treatment. All injections were administered on days 0, 3, 5 and 7 [33]. In the study by Gigante *et al.* platelet rich fibrin matrix (PRFM) was prepared using a commercial kit. A single administration of PRFM was filled in one side of the body while the contralateral injured muscle (control) was left untreated [34].

### Effectiveness of Interventions

Summary of the characteristics of each study is presented in Table 3. The primary outcome in all studies was quantification of muscle regeneration (myogenesis). In two studies this was achieved by immune-histochemical detection of Myogenin and MyoD (markers of muscle regeneration) [33]–[35]. Whereas Wright-Carpenter *et al.* used Ki-67 marker as indicator of satellite cells proliferation [30]. Only one study assessed muscle functional recovery in addition to the tests mentioned above. Hammond *et al.*, measured maximal isometric contraction of the dorsiflexors before injury and again four minutes after injury (to measure force lost because of injury). Maximal isometric torque was retested at days 3, 5, 7, 14 and 21 after injury [33]. All studies demonstrated significantly greater muscular regeneration in the intervention group than controls [33]–[34]. In addition, Wright-Carpenter *et al*. demonstrated increased in satellite cells activation as early as 30 & 40 hours after the ACS therapy [32]. Accordingly higher number of central nucleated myofibers (larger diameter fibres) was found in PRP and ACS treated rodents. Interestingly Hammond *et al.* found PRP therapy had little effect on single-repetition injury protocol. Conversely, in the multiple-repetition protocol, PRP treatment significantly improved contractile function and effectively shortened the time to full recovery from 21 to 14 days [33].

### Studies Methodological Quality

Our extensive search only resulted in a single human pilot clinical trial; the particular studies demonstrated several limitations including the use of retrospective data of athletes treated with Actovegin/Traumeel as controls and unreported baseline participant’s information. Using the PEDro scale this study score 4 of maximal 10, represented a low quality study. Surprisingly our search did not find any human cross-sectional or case control study with regard to the use of PRP for muscle injuries. Furthermore only three *in vivo* laboratory studies were retrieved by the search.

## Discussion

From the available evidences presented currently there is no randomised controlled trials available and the number of well-designed CT on the use of PRP therapy for muscle injury is limited. Only one human study was identified while the remaining three studies were *in vivo* laboratory studies on rodents. All three *in vivo* studies reported histological acceleration of muscle recovery/healing in the intervention group. Only one study however, demonstrated concurrent early functional muscle recovery [34].

Three studies, including the pilot CT demonstrated significantly higher concentration of certain growth factors in the injectables prepared (PRP and ACS) [32], [33], [36]. Wright-Carpenter *et al*, attributed the efficacy of ACS to the significant increased in FGF-2 concentration (750%) [36]. The effect of concomitant significant increased in other growth factors including TFG-β1 (31%) levels were not discussed. Furthermore histological analysis in these studies focussed only on new myofibers regeneration without exploring on the amount of fibrosis accompanying muscle healing. Analysing the amount of scar formation is as important since increased fibrosis has been associated with TFG-β1 and contributes to risk of reinjuries [37]. Recent laboratory study demonstrated more potent effect of PRP in accelerating functional muscle recovery when combined with substance that neutralising effect of TFG-β1 [38]. More clinical studies are required to explore the individual and collective effects of the various growth factors and cytokines within PRP on muscle recovery. Such information is useful in improving treatment efficacy and safety by enhancing desirable and blocking unwanted effects [39].

With regards to functional recovery, Gigante *et al.* reported rodents induced with multiple repetitions (small strain) demonstrated significantly shorter time to achieve functional muscle recovery in the PRP treated group compared to controls. The PRP therapy was less effective for treating larger muscle strain (single repetition induced). This observation suggests that PRP therapy may not be equally effective for all type of muscle injuries.

In the pilot study of professional sportsmen with second-degree muscle strain, athletes treated with repeated injections of ACS reported earlier subjective readiness to resume activities at competitive level than those treated with Actovegin/Traumeel.

### Applicability of Evidence

Professional sportsmen receiving ACS therapy reported significantly faster subjective impression of readiness to resume activities at competitive level than controls. The methodological quality of this CT scored 4/10 on the PEDro scale and rated as poor quality. Several limitations were detected in this pilot study, including lack of randomisation, absence of concealment of treatment allocation, absence of baseline data characteristics between subjects in both groups, and lack of blinding (subjects, therapists or assessors). Further, inadequate methodological approaches in trials were shown to be associated with bias [40]. Results from this trial must be interpreted with great cautious.

A more objective assessment such as validated symptoms’ questionnaires and functional assessment (isokinetic strength assessment) may be useful in these circumstances as it may establish a more objective and standard assessment of readiness to participate in pre-injury level activities.

The methodological quality of the *in vivo* studies varies in several aspects. Significant acceleration of muscle regeneration was reported by all three studies. Concomitant improvement in contractile function and faster time to full recovery was shown in animals with small strain injury treated with ACS injection [31]. Whether similar cellular changes observed in these *in vivo* studies would occur in humans remains unanswered. Replicating such study in humans will be challenging, in view of substantial ethical consideration on the need to biopsy the injured muscle especially among competitive athletes. Furthermore, the importance and difference between cellular versus functional recoveries should be considered. There are limitations from this review. Only peer-reviewed papers published until 2012 and in English language were included in the data extraction, hence a possibility of selection bias. In addition, even though the searches are done thoroughly through multiple major databases with cross-referencing; there is a possibility that some papers were not included due to the inclusion criteria used for this current review.

## Conclusion

In conclusion, there are limited studies on the effects of PRP therapy on muscle recovery. Our review identified only a single pilot human controlled trial [34] and three *in vivo* laboratory studies [30]–[32]. All the three *in vivo* studies reported histological evidence of significant acceleration of muscle healing in the experimental groups (ACS, PRP and PRFM). Whether such findings can be translated into humans, remain to be answered, as valuable information from robust human controlled trials is still not available at this moment.

Our review found no evidence with good methodological quality to suggest that PRP therapy is effective in accelerating muscle recovery after injury.

## Supporting Information

Checklist S1 **PRISMA Checklist.**
(PDF)
